# Latent variable sequence identification for cognitive models with neural network estimators

**DOI:** 10.3758/s13428-025-02794-0

**Published:** 2025-08-28

**Authors:** Ti-Fen Pan, Jing-Jing Li, Bill Thompson, Anne GE Collins

**Affiliations:** 1 Department of Psychology, University of California, Berkeley, USA; 2 Helen Wills Neuroscience Institute, University of California, Berkeley, USA

**Keywords:** Computational cognitive models, Intractable likelihood, Artificial neural networks, Latent variables

## Abstract

Extracting time-varying latent variables from computational cognitive models plays a key role in uncovering the dynamic cognitive processes that drive behaviors. However, existing methods are limited to inferring latent variable sequences in a relatively narrow class of cognitive models. For example, a broad class of relevant cognitive models with intractable likelihood is currently out of reach of standard techniques, based on maximum a posteriori parameter estimation. Here, we present a simulation-based approach that leverages recurrent neural networks to map experimental data directly to the targeted latent variable space. We first show in simulations that our approach achieves competitive performance in inferring latent variable sequences in both likelihood-tractable and intractable models. We then demonstrate its applicability in real world datasets. Furthermore, the approach is practical to standard-size, individual data, generalizable across different computational models, and adaptable for continuous and discrete latent spaces. Our work underscores that combining recurrent neural networks and simulated data to identify model latent variable sequences broadens the scope of cognitive models researchers can explore, enabling testing a wider range of theories.

Researchers often use time-varying latent variables from computational cognitive models to capture the across-trial dynamics of cognitive processes. These variables provide trial-by-trial predictors of internal states, enabling investigation into underlying mechanisms and individual differences (Katahira & Toyama, 2021). For instance, psychologists apply state-space models (e.g., hidden Markov models) to infer time-varying attentive states, offering better explanations for noisy behaviors (Ashwood et al., 2022; Li et al., 2024). In neuroscience, model-based analyses commonly link neural activity to latent variables derived from computational cognitive models, illuminating how the brain supports cognition (Cohen et al., 2017). Notably, reward prediction errors (RPE) extracted from a reinforcement learning (RL) model have been found to correlate with ventral striatum activity in human functional magnetic resonance imaging (fMRI) (O’Doherty et al., 2007) studies, as well as phasic activity of dopamine neurons in non-human animals (Eshel et al., 2016).

The traditional method for extracting trial-by-trial latent variables from experimental data consists of two steps (Wilson & Collins, 2019): first, identify the best-fitting model and model parameters; second, infer the latent variables by running the computational model over participants’ experienced sequences of stimuli and actions with the best-fitting model and model parameters. However, both steps limit the type of models that can be considered. First, best-fitting parameters may be difficult to obtain in a large subspace of relevant cognitive models. Indeed, researchers typically use likelihood-dependent methods like maximum likelihood estimation (MLE) (Myung, 2003), maximum a posteriori (MAP) (Cousineau & Helie, 2013) or hierarchical Bayesian modeling (Baribault & Collins, 2023). These methods are unsuitable for models whose likelihoods are analytically intractable, lacking a closed-form mathematical solution, or computationally intractable, requiring excessive time to compute (Lenzi et al., 2023). Second, latent variables may be complex to infer even when best-fitting parameters are known, requiring custom design of statistical tools specific to a model and experiment at considerable cost (Findling et al., 2021; Ashwood et al., 2022). Due to these limitations, testing a broader class of theories is significantly slowed down by the need for researchers to develop customized statistical approaches that are not generalizable to broader computational models (Ashwood et al., 2022; Escola et al., 2011).

Most computational models with intractable likelihoods can be simulated, making simulation-based inference (SBI) a powerful approach to bypass the likelihood computation (Busetto Alberto et al., 2013). SBI methods, particularly when combined with artificial neural networks (ANNs), have proven effective in both model identification and parameter recovery across various computational models. ANNs excel in handling high-dimensional data and enabling amortized inference after training. These neural-based SBI methods are primarily based on Bayesian inference, aiming to approximate likelihood (neural likelihood estimation, NLE) (Boelts et al., 2022) or the posterior (neural posterior estimation, NPE) (Radev et al., 2020) (see the recent review Zammit-Mangion et al., 2024). Recently, the approach consisting of training neural networks to map data to parameter point estimates has shown promising avenues in a variety of applications (Lenzi et al., 2023; Sainsbury-Dale et al., 2023). Sainsbury-Dale et al. (2024) conceptualized this method by connecting it to classic Bayes estimators, which we refer to as neural point estimators here.

However, the extraction of time-varying latent variables in likelihood intractable models is still under-explored (Schumacher et al., 2023). Existing SBI methods focus mainly on parameter recovery or model identification (Gloeckler et al., 2024; Zammit-Mangion et al., 2024). Although SBI methods help recover the time-invariant model parameters from individual participants’ data, approximating time-varying latent variable sequences often proves challenging. This is especially true in models with sequential dependencies across trials, where sampling methods like sequential Monte Carlo (SMC) (Doucet et al., 2000; Gordon et al., 1993; Samejima et al., 2003) can be computationally expensive when reconstructing latent variables (Ghosh et al., 2024).

Here, we propose an approach that aims to identify **La**tent variable **Se**quences with ANNs, which we call "LaseNet". LaseNet is built on neural point estimators, given their success in applying to computational cognitive models with sequential dependencies and intractable likelihoods (Rmus et al., 2024). We use simulated datasets to train an ANN, which learns a direct mapping between a sequence of observable variables (e.g., the participant’s actions or received outcomes) and the targeted latent variable space (e.g., the participant’s reward expectation or subjective rule choice). We begin by outlining the problem formalism and cognitive models in the following section, followed by a detailed introduction of the LaseNet in Sect. “Method”. In Section “Synthetic dataset results”, we show that LaseNet infers time-varying latent variables that are close to synthetic ground truth for a variety of computational cognitive models and task environments. We highlight LaseNet’s real-world applicability in Section “LaseNet infers latent variable sequence in real data”: using experimental data from a real mice dataset, our approach successfully infers both discrete and continuous latent variables compared to likelihood-dependent estimations. Finally, we discuss related work and the benefits and limitations of LaseNet.

## Problem formulation

Suppose that there is a latent variable model that produces a series of observable variables across trials: $Y=(y_{1}, y_{2}...y_{T})$; and unobservable latent variables $Z \!=\! (z_{1}, z_{2}... z_{T})$ conditioned on a set of model parameters $\theta$, where *T* denotes the number of trials. Here, we define **model parameters** as time-invariant (fixed across trials) and **latent variables** *Z* as time-varying and conditioned on the model parameters (see examples below). We can then describe the generation process for the time-varying latent variables as follows:

1 $$\begin{aligned} z_{t+1}\sim f(z_{t}, y_t, \theta _f),\; \; \; \; \, \; y_t\sim g(z_{t}, \theta _g) \end{aligned}$$

where *f* (parameterized by $\theta _f$) and *g* (parameterized by $\theta _g$) are density functions describing the evolution of latent model variables and the generation of conditional observations, respectively. This generative process adheres to the Markov property, wherein the next state depends solely on the current state; note that this is not a restrictive property in practice, as the latent variable $z_t$ can be defined with sufficient complexity to encapsulate long-range historical information (e.g., a continuous variable summarizing past history). Our goal is to infer the unobservable latent variables *Z* at each time point, conditioned on the observed sequence *Y*. Standard approaches, such as MLE, typically involve estimating the model parameters $\theta$ first and then deriving the latent variables *Z* conditioned on the inferred model parameters:

2 $$\begin{aligned} \widetilde{\theta } = \arg \max _{\theta } \, P(\theta \mid Y) \end{aligned}$$

3 $$\begin{aligned} \widetilde{Z} = \arg \max _{Z \in \text {K}} \, P(Z \mid \widetilde{\theta }, Y) \end{aligned}$$

where K denotes the possible values in the latent variable space, which can be either continuous $Z_{c}$ or discrete $Z_{d}$. $\theta$ includes $(\theta _f, \theta _g)$. Alternatively, iterative methods like the Expectation-Maximization (EM) algorithm can be used to jointly estimate both *Z* and $\theta$ by updating each one in turn until the estimates converge (Huys et al., 2015). In contrast, our proposed method bypasses the explicit optimization of $\theta$ and the need to compute the likelihood of the data. Instead, it directly aims to find the latent variable sequence *Z* that maximizes its marginal posterior probability given only the observations *Y*, simplifying the objective to: $\widetilde{Z} = \arg \max _{Z \in \text {K}}\, \, P(Z \mid Y)$.

### Task environments and computational cognitive models

We illustrate our approach with representative examples of sequential learning and decision-making tasks, where biological agents (e.g., humans, mice) are assumed to be in a state (for example, defined by an observable stimulus), select actions, and may observe outcomes. Examples of stimuli include specific images or abstract features characterized along a specific dimension, such as the orientation of a grating. Agents’ choices typically correspond to pressing one of multiple keys or levers; subsequently, feedback outcomes are typically points for humans or water for mice. Computational cognitive models are simple algorithms that operate over a few variables (typically $<10$); they instantiate specific hypotheses about the information flow and provide quantitative predictions about behavioral data. To illustrate our technique, we use three representative families of cognitive models as follows:

#### Reinforcement learning (RL) models

RL cognitive models, such as delta rule or Q-learning (Eckstein et al., 2022; Niv et al., 2012; Sutton et al., 2018) assume that an agent tracks the Q-value of actions, and uses these Q-values to inform action selection on each trial. After each trial’s outcome, the model updates Q-values by first computing the reward prediction error (RPE), denoted by $\delta$, as the discrepancy between the expected and the observed values, and then adjusting the Q-value of the chosen action *a* with RPE scaled by a learning rate $\alpha$ (Sutton et al., 2018):

4 $$\begin{aligned} \begin{gathered} \delta _t = r_t - Q_t(a_t) \\ Q_{t+1}(a_t)=Q_t(a_t) + \alpha \,\delta _t \end{gathered} \end{aligned}$$

One example of a time-varying latent variable of interest to model-based analyses (Cohen et al., 2017) is Q-values ($Z_{c}$), which are inferred from observable rewards, stimuli, and actions (*Y*); here the model parameters are $\alpha \,(=\theta _f)$ and $\beta \,(=\theta _g)$. This model is typically likelihood *tractable*, providing a baseline for standard approaches. In addition, we explore a hierarchical extension of this model, where choices are governed by higher-level rules that are not directly observable, making the model’s likelihood *intractable* (more details in “Benchmark settings”). This allows us to investigate latent rule inference over discrete latent states ($Z_{d}$).

#### Bayesian generative models

We consider a variant of hierarchical Bayesian generative models that incorporates noisy inference following Weber’s law (Fechner, 1860; Findling et al., 2021), positing that computational imprecision scales with the distance between posterior beliefs in consecutive trials (*t* and $t+1$). This model is likelihood-intractable; however, a custom statistical approach was developed, providing a baseline for our approach. Our study aims to infer both the agents’ discrete latent state ($Z_{d}$) and the distance ($Z_{c}$) in posterior beliefs across trials given observable rewards, stimuli, and actions (*Y*).

#### Hidden Markov models (HMMs)

We use a cognitive model based on the Bernoulli generalized linear model (GLM) observations (GLM-HMM) (Ashwood et al., 2022; Calhoun et al., 2019; Escola et al., 2011). Again, this model is likelihood-intractable, but a custom statistical approach provides a benchmark. Our example target output is the HMM hidden states ($Z_{d}$) given rewards/stimuli and actions (*Y*). See Appendix A for more detailed descriptions of the cognitive models.

## Method

### RNN-based inference for time-varying latent variables

**Fig. 1:**
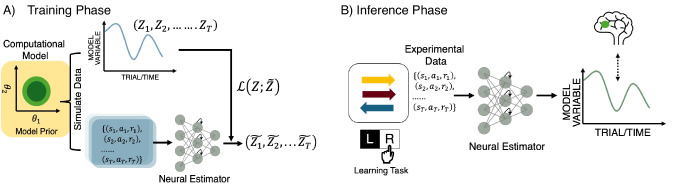
Schematic of the LaseNet method: **A** The network is trained with a simulated dataset to predict time-varying latent variables derived from a computational cognitive model. The input may include a time series of simulated stimuli, actions, and rewards. Corresponding latent variables are used as training targets. **B** At inference time, the trained network predicts the latent variables for experimental data from biological agents where ground truth is unknown. The extracted latent variables are commonly used in relating behavior to brain signals

Our proposed method relies on a two-phase approach, similar to standard ANN-based SBI methods: a training phase and an inference phase (Fig. 1). During the training phase, we first create a synthetic dataset by simulating the computational cognitive model of interest on the target experimental task. An artificial neural network (*LaseNet*) is then trained on this synthetic dataset using model-simulated observable data $\textbf{Y}$ as input and a series of model-derived latent variables $\textbf{Z}$ as output. During the inference phase, the trained LaseNet takes the observable experimental data as input to infer a sequence of unobservable latent variables.

#### Architecture

**Fig. 2:**
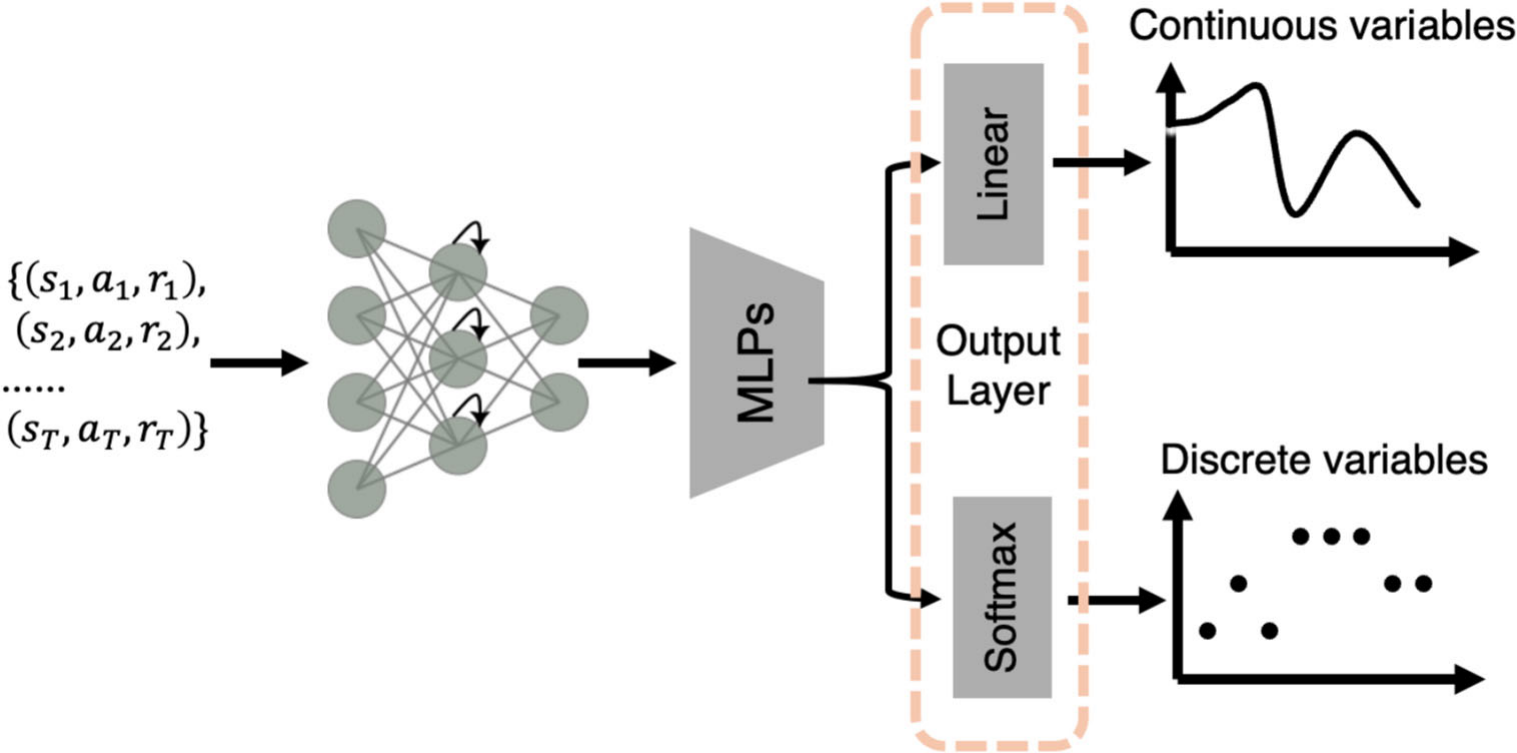
Network structures: The building blocks of LaseNet consist of one recurrent neural network (RNN) followed by two layers of multilayer perceptrons (MLPs). Depending on the goal of variable inference, we add either one or two different output layers to predict discrete and/or continuous latent variables; they receive the same embeddings from MLPs as input

LaseNet aims to learn a mapping between the observable variable space *Y* and latent variable space *Z*. To learn this relationship, the structure of LaseNet is composed of two components: a bidirectional recurrent neural network (bi-RNN) layer (Schuster & Paliwal, 1997) followed by multilayer perceptrons (MLPs). The MLP layer has a pyramidal shape (Fig. 2), decreasing layer width from the largest to the output dimension. After the bi-RNN layer, the number of units of each layer is half of the previous layer (see Table 2 for detailed specification).

The building blocks of bi-RNN are gated recurrent units (GRU) (Cho et al., 2014). Bidirectionality enables the network to learn embeddings from both past and future history (Graves & Schmidhuber, 2005). This is advantageous for latent variable/state inference compared to a unidirectional GRU (performance in Appendix Table 3). Since the next latent state is conditioned on the current, future information can inform the current state, which is also influenced by the past. A summary embedding *S* is thus yielded by concatenating the past and future embeddings. We can represent the learned summary *S* at each time point $y_t$ as:

5 $$\begin{aligned} S(y_{t}) = \left\langle \overrightarrow{\psi }(\left\{ y \right\} _{i=1}^{t}),\overleftarrow{\psi }(\left\{ y \right\} _{i=t}^{T}) \right\rangle \end{aligned}$$

where function $\overrightarrow{\psi }$ (forward pass), $\overleftarrow{\psi }$ (backward pass) transform a time series $(y_{1}, y_{2}...y_{T})$ to a lower-dimensional embedding space. Following the bi-RNN, MLPs map the summary embeddings to the targeted latent variable space. Let $\phi$ denote a universal function approximator. We can describe the estimated latent variable for each time point as $\tilde{z}_t = \phi (S(y_{t}))$. When training LaseNet, the main objective is to find a set of neural network parameters (i.e., weights or biases) that minimizes the loss between true and estimated latent variables: $\mathcal {L}(\textbf{Z};\tilde{\textbf{Z}})$.

**Table 1:** Summary of five computational cognitive models. a, r, and s denote actions, rewards, and stimuli, respectively. For example, 7-d s indicates that the stimulus input is coded as a seven-dimensional one-hot input vector

Name	# of $\theta$	Input of LaseNet (*Y*)	Output of LaseNet (*Z*)	Tractable
4-P RL	4	$\mathbb {R}^{2}$ [a, r]	$\mathbb {R}^{1}$ Q-value	Yes
Meta RL	9	$\mathbb {R}^{2}$ [a, r]	($\mathbb {R}^{1}$ , $\mathbb {R}^{2}$) (Q-value, attentive state)	Yes
HRL	2	$\mathbb {R}^{5}$ [a, r, 3-d s]	($\mathbb {R}^{1}$ , $\mathbb {R}^{3}$) (Q-value, chosen cue)	No
Weber-imprecision	3	$\mathbb {R}^{9}$ [a, r, 7-d s]	($\mathbb {R}^{1}$ , $\mathbb {R}^{24}$) (distance, chosen mapping)	No
GLM-HMM	21	$\mathbb {R}^{3}$ [a, r, 1-d s]	$\mathbb {R}^{3}$ attentive state	No

#### Output layers

By changing the output layers, our architecture is adaptable to both continuous and discrete latent variables. For a continuous latent model variable space (e.g., Q-value), we used a linear activation with mean-squared error (MSE) loss. For a discrete latent model variable space (e.g., chosen strategy), we used a softmax activation function in the output layer, with a cross-entropy loss. To predict both types of latent spaces at once, we added two output layers for each type after the MLP layers (Fig. 2).

#### Details of neural networks Training

We used rectified linear unit (ReLU) as an activation function in all MLPs layers. The trainings were run on Nvidia L4 GPUs, each equipped with 25 GB of memory, and required at most 30 minutes to complete (Appendix Table 4). Network parameters were randomly initialized and optimized by Adam, with a learning rate of $3*10^{-4}$. Each network was trained with at most 600 epochs and a batch size of 128. To avoid overfitting, we used 35 epochs as our early stopping criteria based on validation loss. Hyperparameters were fine-tuned using Bayesian optimization algorithms (Appendix Table 2) with Bayesian optimization algorithms (Bergstra et al., 2013) applied to the validation set. We allocated 10% of the training data as the validation set in fine-tuning.

### Benchmark settings

#### Cognitive models and tasks

(Table 1) We evaluated LaseNet against five computational cognitive models and task environments. We first evaluated two tractable models: a four-parameter reinforcement learning model **(4-P RL)** (Zou et al., 2022), and a meta reinforcement learning model with dynamic noise **(Meta RL)** (Li et al., 2024). We tested LaseNet’s ability to infer both continuous and discrete variables from these cognitive models on a two-armed bandit learning task with probabilistic reversal (Cools et al., 2002). We then validated LaseNet in three likelihood intractable models:

- Hierarchical reinforcement learning **(HRL)** model performing a novel dynamic decision making task (Rmus et al., 2024). In this task, participants observe three colored arrows, each pointing left or right (Fig. 1B). To earn rewards, participants choose a direction (left/right) based on the correct arrow, which changes unpredictably. This task is hierarchical because the correct choice (left/right) depends on a higher-level rule tied to arrow color. We assume that agents use a simple RL process to track arrow values and select a discrete arrow, before deciding on a side. Under these assumptions, the model is intractable, because the internal “arrow rule” selected by agents is unobservable and conditions on Q-value updates. It is one of the targets of latent variable estimation.
- Weber-imprecision model (a variant of Bayesian generative model) (Findling et al., 2021) performing a stimulus-action learning task in a volatile environment (Collins & Koechlin, 2012). In this task, participants learn to match three stimuli to four different actions. The volatile environment shifts between distinct sets of stimulus-action mappings with a probability of 3%, requiring participants to continuously adapt to new stimulus–action associations. These mappings (or task sets) are latent states, with 24 possible mappings/latent states in total.
- 3-state GLM-HMM (Ashwood et al., 2022) performing a standard two-choice perceptual decision-making task (Laboratory et al., 2021).

#### Dataset

For each LaseNet estimator, we simulated at most 9000 pairs of $({\textbf {Z}},{\textbf {Y}})$ with 720 trials in each pair as training data (representing a standard cognitive task duration for a real biological agent). We hold out 10$\%$ of training data as the validation set to fine-tune the hyperparameters. We simulated an additional 1000 unseen pairs of $({\textbf {Z}},{\textbf {Y}})$ as testing data.

#### Other estimators

As a comparison against LaseNet, we tested four commonly used likelihood-dependent estimators: MLE, MAP (Wilson & Collins, 2019), expectation–maximization (EM) (Dempster et al., 1977), and sequential Monte Carlo (SMC) (Chopin et al., 2013; Doucet et al., 2000) on the same dataset. Two steps are required for researchers to recover latent variables with likelihood-dependent estimators. The first step is to find the best fitting model parameters $\theta$ given observable data **Y** by either maximizing a likelihood function $P(\textbf{Y}\mid \theta )$ or posterior probability $P(\textbf{Y}\mid \theta )\,\,P(\theta )$. In the second step, we used the best-fitting parameters $\tilde{\theta }$ and observable data **Y** to derive the latent variables **Z**. We then approximate latent variables from the targeted cognitive models **f** with $\tilde{\theta }$ and **Y** as input:

$\tilde{\textbf{Z}} \approx \textbf{f}(\textbf{Y};\tilde{\theta })$. Note that throughout (except stated otherwise, e.g., in Appendix “Model misspecification”), we assume that the best model has been identified through a model comparison and validation procedure (Wilson & Collins, 2019). This step can be performed for likelihood intractable models using neural-based approaches similar to LaseNet (Rmus et al., 2024). See Appendix B for more detailed descriptions of the methods.

## Synthetic dataset results

### Evaluation metrics

For all the metrics in this work, results were averaged across trials, where marker and error bars represent the mean and 2 standard deviations over all test samples.

#### Root mean-squared error

RMSE measures the difference between true ($Z_c$) and predicted continuous latent variables ($\hat{Z}_c$)(e.g., Q-values) from the estimators. RMSE across trials 1 to *T* is defined as:

6 $$\begin{aligned} \text {RMSE}\,(\mathbf {Z_c},\hat{\textbf{Z}}_\textbf{c})= \sqrt{\frac{1}{T}\sum _{i=1}^{T}(z_{i} - \hat{z}_{i})^{2}} \end{aligned}$$

#### Negative log loss

NLL measures the predicted probability $\textbf{p}$ from the estimators based on ground-truth discrete labels. We use NLL to quantify the performance of inferring discrete latent variables (e.g., attentive states and chosen cues). NLL across trials 1 to *T* and a set of *M* labels is defined as:

7 $$\begin{aligned} \text {Log Loss}\,(\mathbf {Z_d},\textbf{P}) = -\frac{1}{T}\sum _{i=1}^{T}\sum _{j=1}^{M}z_{ij} \log (p_{ij}) \end{aligned}$$

#### Accuracy

We computed the balanced accuracy (Brodersen et al., 2010) that avoids inflated performance estimates on imbalanced datasets. It is the macro-average of recall scores per state. Thus, for balanced datasets, the score is equal to accuracy. We used balanced accuracy to quantify the performance of inferring discrete latent variables by taking the state with the highest probability as the predicted state. Balanced accuracy is defined as:

8 $$\begin{aligned} \text {Balanced Accuracy} = \frac{1}{2}\left( \frac{\text {TP}}{\text {TP} + \text {FN}} + \frac{\text {TN}}{\text {TN} + \text {FP}}\right) \end{aligned}$$

where TP is true positive, TN is true negative, FP is false positive, and FN is false negative.

### Tractable models

We tested LaseNet using synthetic datasets generated from two tractable models within the RL model family. Maximum likelihood estimation (MLE) was used as a benchmark for comparison.

#### 4P-RL

**Fig. 3:**
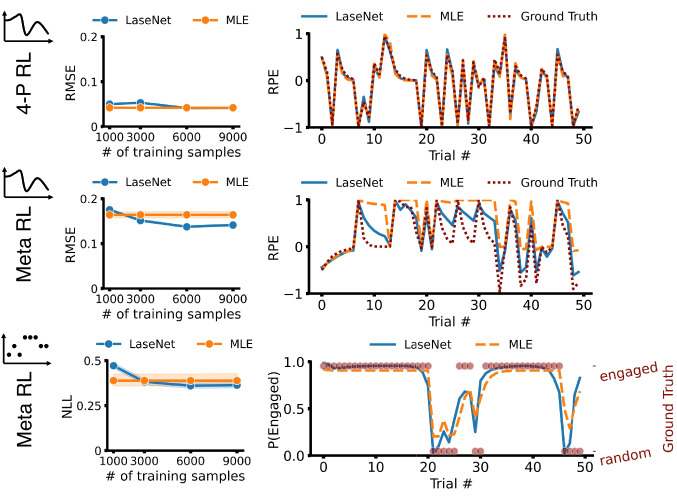
Performance on synthetic dataset: tractable models. The *top row* shows a strong agreement between LaseNet and MLE in the 4P-RL model. The *middle* and *bottom rows* present the results from the Meta RL model, showing that LaseNet identifies both continuous (Q-values) and discrete (engaged vs. random) latent variables with comparable precision but reduced variance (narrower shaded regions) compared to MLE. The right column displays example time series plots for one simulated participant from each model. Note that the reward prediction error (RPE) is obtained by subtracting estimated Q values from rewards

The 4P-RL model is simulated in a two-choice probabilistic reversal learning environment and follows the same Q-value update described in “Task environments and computational cognitive models” with minor variants that bring it closer to human behavior (Zou et al., 2022); the policy is a softmax over Q-values: $P(a_i) \propto \exp (\beta Q(a_i))$, with inverse temperature $\beta$ controlling noise in the policy. All further model and experiment details are available in Appendix A. Our target latent variable is the chosen Q-values, representing the subjective reward expectation at each trial. We found LaseNet reaches a similar average RMSE (M = 0.041) to a standard MLE-based approach (M = 0.042) (first row in Fig. 3), showing our approach’s capability to recover latent variables in a simple model and environment. We next tested it in a more complex cognitive model, where we can infer both continuous and discrete latent variables.

#### Meta RL

Meta RL with a dynamic noise model shares a similar Q-value update policy as 4-P RL. The major difference is that the model assumes a participant has two latent attentive states: engaged and random. The transition from one latent state to the other is controlled by a hidden Markov process (Li et al., 2024). Two time-varying latent variables are inferred here: chosen Q-values (continuous latent space) and two attentive states (discrete latent space). We found that the RMSE of LaseNet (M = 0.141,   SD = 0.038) is slightly lower with less variance compared to MLE (M = 0.164,   SD = 0.108)(last two rows in Fig. 3), after training with 6k simulated participants. Moreover, in identifying attentive states, LaseNet had a slightly lower (better) NLL (M = 0.363) than MLE (M = 0.388). (last row in Fig. 3), which may be due to parameter recovery issues with MLE (detailed in Appendix B).

### Intractable models

In this section, we assessed LaseNet using three distinct intractable models: the HRL model (RL-based model), the Weber-imprecision model (Bayesian generative model), and the GLM-HMM (state-space model). For comparison, we used sequential Monte Carlo (SMC) and expectation-maximization (EM), two widely used methods for approximating likelihoods and inferring time-varying latent variables in intractable cognitive computational models.

**Fig. 4:**
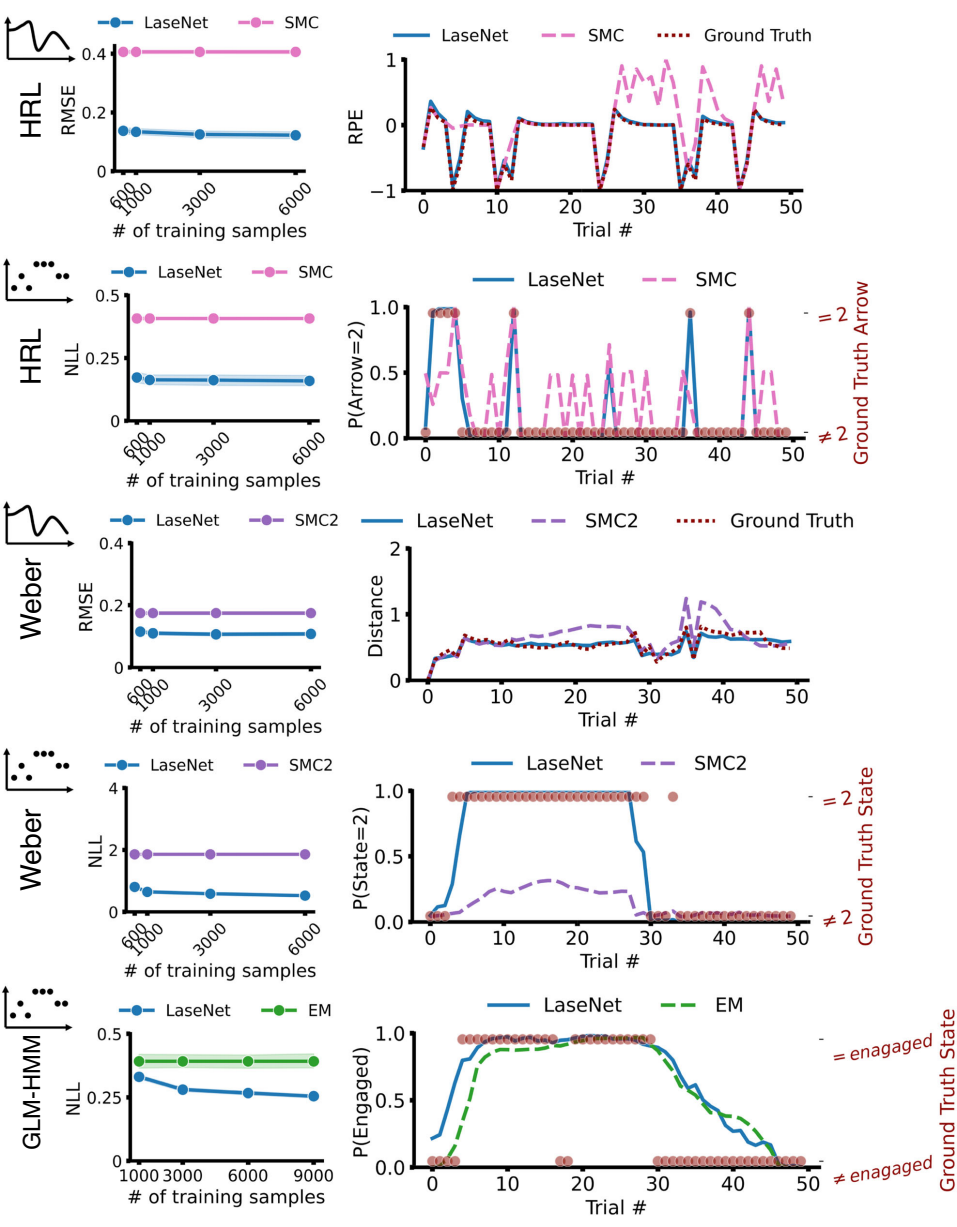
Performance on synthetic dataset: intractable models. The *left column* presents quantitative metrics across the three models, showing that LaseNet achieves lower (better) RMSE and NLL compared to other estimators. The *right column* shows trial-by-trial predictions from all estimators for an example agent. Overall, LaseNet demonstrates closer alignment with the ground truth

#### HRL

The model assumes that participants track the value of each arrow and choose between the arrows noisily: $P(arrow) \propto \exp (\beta \;Q_{t}(arrow))$. Crucially, the chosen arrow is not observable, but only conditions which side the participant selects (which is the observed action). To compute the likelihood, we need to know which arrow the participant internally selected and update the corresponding Q-value; but because the internal arrow choice is unobservable, we need to marginalize over potential arrow choices over all the past trials to compute the current trial’s likelihood, and this marginalization is computationally intractable as the number of trial increases (See Appendix A for more details). The target latent variables include the selected Q-values (representing the value of the chosen arrow) and the chosen arrow. We used SMC, specifically particle filtering, to recover these time-varying latent variables. To isolate the performance of latent variable recovery, we assumed the model parameters were known when applying SMC, thereby bypassing the parameter recovery step. Note that this approximation should confer a benefit to the SMC approach compared to ours. As shown in the top two rows of Fig. 4, LaseNet outperformed SMC. For Q-values identification, LaseNet achieved an average RMSE of 0.122 compared to 0.405 for SMC. In discrete latent cue identification (arrow selection), LaseNet reached 92.3% accuracy, substantially surpassing SMC’s 71.5%. Predicted cues were determined by selecting the cue with the highest probability from the estimators’ output.

**Fig. 5:**
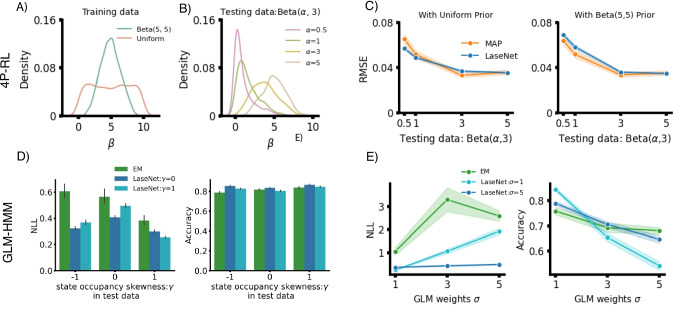
Performance on misspecified priors. The *top row* shows the impact of training with a misspecified prior of the model parameter $\beta$ in a 4-P RL model. **A** We compared two LaseNets trained with different prior distributions: a beta distribution and a uniform distribution. **B** Four test datasets generated from four different distributions of parameter $\beta$ are evaluated. **C** Training with a uniform distribution has more robust performance across different test datasets; the bottom row summarizes the impact of training with misspecified priors in a GLM-HMM model. **D** Three latent state distributions with positive (+1), no (0), and negative (-1) skewness $\gamma$ are evaluated. LaseNet trained with a uniform distribution (no skewness) outperforms both LaseNet trained with positive skewness and EM here. **E** Adjusting the $\sigma$ (noise level) in GLM weights in GLM-HMM models reveals that LaseNet is more robust when trained with a noisier dataset (i.e., higher $\sigma$)

#### Weber-imprecision

In a stimulus-action learning task, the model assumes that participants are more likely to change stimulus-action mapping (latent state) when the distance between posterior beliefs in consecutive trials is greater. Behavioral noise $\epsilon _t$ is modeled as a random variable uniformly distributed between 0 and $\mu + \lambda d_t$, where $d_t$ is the distance between posterior beliefs in trial $t-1$ and *t*. The parameters $\mu$ and $\lambda$ quantify Weber-imprecision. The target latent variables are the posterior belief distance $d_t$ and the chosen state/mapping. We compared LaseNet with SMC2 (Chopin et al., 2013), as used in Findling et al. (2021). Consistent with the HRL benchmarking, we assumed known model parameters for SMC2. Results in Fig. 4 (middle two rows) demonstrate that LaseNet outperformed SMC2, with an average RMSE of 0.107 for LaseNet compared to 0.174 for SMC2. LaseNet achieved 54.7% accuracy in latent state identification, while SMC2 reached 45%, far above the chance level of 4% (1/24). Additionally, LaseNet exhibited significantly lower NLL, indicating higher precision in predicting state probabilities (fourth row; right column in Fig. 4).

#### GLM-HMM

A 3-state GLM-HMM is used to capture three attentive states: engaged, biased-left, and biased-right (Ashwood et al., 2022) in a two-choice perceptual decision-making task. This model comprises three independent Bernoulli GLMs, each conditioned on the participant’s current discrete strategy state. Each GLM is characterized by a weight vector that defines how inputs are integrated into decision policies specific to that state. We trained LaseNet to predict the time-varying attentive states and compared its performance to the approximate EM algorithm, as used in Ashwood et al. (2022). We found that LaseNet achieved a lower average NLL of 0.255 compared to EM’s 0.392, indicating superior performance in predicting state probabilities (last row of Fig. 4). While the accuracy of both methods was comparable, LaseNet slightly outperformed EM, achieving 84.3% accuracy versus EM’s 83.2%.

### Prior misspecification

We examined the impact of misspecified model parameter priors with LaseNet and the likelihood-dependent methods in one tractable (4-P RL) and one intractable (GLM-HMM) model. For likelihood-dependent methods, model parameter priors are standard Bayesian priors applied during the model fitting procedure (i.e., MAP or EM). In contrast, LaseNet employs these priors to guide the sampling of model parameters (e.g., $\alpha$, $\beta$) for generating its training data. While this latter use is not Bayesian, it serves a similar role of constraining the pattern of latent variables that LaseNet is likely to infer after training. Overall, LaseNets trained with the dataset generated from a less biased prior distribution resulted in a more robust performance. All LaseNets for 4P-RL were trained with 22k simulated participants and with 9k simulated participants for the GLM-HMM.

#### 4P-RL

softmax $\beta$ parameter in 4-P RL controls the randomness of a participant’s action: higher beta results in more deterministic actions. We evaluated two priors of $\beta$: a Beta ($\alpha$=5,$\beta$=5) prior (green line in Fig. 5A) and a uniform prior within an empirical range (orange line in Fig. 5A). We trained two LaseNet estimators with datasets generated from these two priors, respectively. We tested the performance of LaseNet and MAP estimators with four different $\beta$ priors (red, light green, yellow, and brown lines in Fig. 5B). We found that the LaseNet trained with a uniform prior had lower RMSE (0.044) across different $\beta$ priors (Fig. 5C).

**Fig. 6:**
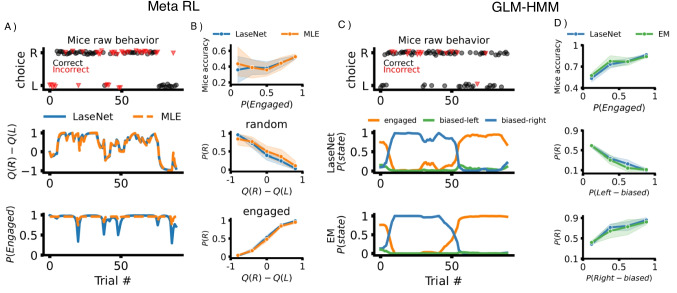
Validation of LaseNet application on real mice data. **A** *Top*: raw behavioral data of one example mouse in the dynamic foraging dataset. Each *dot* represents a single trial; the *y*-axis indicates if the mouse went rightward or leftward on the trial. *Middle*: estimated Q-values difference. *Bottom*: latent engaged probability trial-by-trial in the example mouse. **B** LaseNet and MLE identify similar relationships between the mice’s response accuracy and the probability of rightward choice given estimated latent variables. **C** *Top*: raw behavioral data of one mouse in the IBL dataset. *Middle* and *bottom rows*: state probabilities from LaseNet and EM estimation, respectively, highlighting high agreement of certainty about the mouse’s internal state. **D** LaseNet and EM show high consensus with the mice’s response accuracy and probability of rightward choice given different latent policy states

#### GLM-HMM

Here, we changed the priors of the hidden state distribution (transition matrix) and GLM weights, independently. In state distribution, we tested three skewness $\gamma$ levels: positive (1), negative (-1), and no (0) skewness. No skewness means that each state occupancy is equal (i.e., uniform distribution). We examined two LaseNet estimators: one is trained with positive skewness, and the other is with no skewness in comparison with EM having a positive skewness prior. We showed that LaseNet with equal states prior (no skewness) reaches the highest accuracy and lowest NLL among all (Fig. 5D). Furthermore, in changing the $\sigma$ in the GLM weights with a fixed mean, we found that LaseNet trained with a higher $\sigma$ (noiser) dataset is more robust (Fig. 5E). This suggests our approach is applicable even if there are no strong empirical priors.

## LaseNet infers latent variable sequence in real data

### Meta RL inference in mice dynamic foraging dataset

The dynamic foraging dataset consists of 48 mice’s data collected by Grossman et al. (2022). Each mouse did a two-armed bandits task with dynamic reward schedules. We trained LaseNet to infer the latent attentive state (engaged vs. random), Q-values of left and right actions. In comparison, we adopted MLE with an estimated likelihood function described by Li et al. (2024) as a benchmark. We found that in estimating Q-values, LaseNet had similar results to MLE. However, in attentive state identification, MLE tends to estimate state probability with high certainty (Fig. 6A). In the behavioral analysis (Fig. 6B), we found a similar trend between LaseNet and MLE: mice exhibit higher response accuracy when estimated engaged state probability is higher, and the inferred policy (probability of right choice *P*(*R*) as a function of $Q(R)-Q(L)$) is consistent with the inferred latent states, validating the model assumptions.

### GLM-HMM inference in mice decision making dataset

We used a mice decision making dataset published by the International Brain Laboratory (IBL) (Laboratory et al., 2021). The dataset consists of 37 mice performing a visual detection decision-making task developed in Burgess et al. (2017). We extracted a time series of choice, reward, and stimuli data from mice and fed it into the trained LaseNet for inference. The LaseNet inferred time-varying probabilities for three HMM states (engaged, left-biased, right-biased). In comparison, we used EM fitting procedure described in Ashwood et al. (2022) as a benchmark. We obtained similar results in predicting state probabilities between LaseNet and EM (Fig. 6C). Figure 6D shows a high agreement between LaseNet and EM, the mean absolute difference is 0.027 in mice accuracy, and 0.037 in right choice probability

## Discussion

We proposed a novel method, LaseNet, for learning a mapping between observable data space and a targeted latent variable space using ANNs and synthetic data from cognitive model simulations. Unlike statistical estimators such as maximum likelihood estimation (MLE) or Sequential Monte Carlo (SMC), LaseNet infers latent variables without requiring likelihood computations. Additionally, our approach differs from neural point/posterior estimators: LaseNet focuses on identifying latent variables within computational models instead of recovering model parameters. Our method does not require large data sets, works at the individual rather than group level, and can be applied to any cognitive model that is simulatable within a given experimental framework.

LaseNet offers several advantages over statistical estimators when applied to computational cognitive models. First, it performs comparably to statistical estimators in tractable models but is more robust in cases with many free model parameters (e.g., Meta RL). This robustness stems from its ability to bypass parameter recovery entirely, directly inferring latent variables from observable data. Second, the flexible architecture of LaseNet can handle both continuous and discrete latent spaces, enabling a single network to simultaneously infer both types without performance degradation.

Furthermore, LaseNet excels in identifying latent variables within likelihood-intractable models, eliminating the need for custom and complex statistical estimators that approximate likelihoods, such as SMC or approximate EM algorithms. This makes LaseNet particularly advantageous for researchers working with simple intractable models, such as HRL, as it provides a generic framework for inference without requiring bespoke statistical methods.

When training with a dataset derived from less informative model parameter priors, LaseNet becomes more robust to different prior distributions in the test set, making it more likely to perform well on real participant data. To achieve high performance, the network does not require a large training sample (up to 22K simulated individual agents). It implies that, in a real-world experimental setting, researchers can use LaseNet to fit experimental data without using strong empirical model parameter priors and a high computational budget. Lastly, in comparison to other statistical estimators, LaseNet showed its generalizability to identify latent variables in a wide range of cognitive models (RL-based, HMM-based, and Bayesian generative models).

### Related work

Our framework is inspired by a wide range of studies that used ANN and SBI to overcome the challenges of likelihood computation in intractable models (Cranmer et al., 2020; Radev et al., 2020; Rudi et al., 2022). Specifically, our work focuses on the approach of mapping data to variable point estimates using neural networks. Recent work (Sainsbury-Dale et al., 2024) formalized the neural point estimation within a decision-theoretic framework. These neural point estimators date back to (Chon & Cohen, 1997) and have shown promising results in a variety of fields, including spatio-temporal forecasting (Zammit-Mangion & Wikle, 2020), spatial fields (Gerber & Nychka, 2020; Lenzi et al., 2023), and time-series (Rmus et al., 2024).

RNNs have been widely used in the processing of time-series data, a prevalent format in computational cognitive models. While earlier studies have employed RNN-based models as cognitive models themselves (Dezfouli et al., 2019; Miller et al., 2024), recent research has taken a different approach, leveraging RNNs as tools for parameter recovery and model identification in computational cognitive frameworks (Ger et al., 2024a, b; Rmus et al., 2024). For example, Russek et al. (2024) successfully showed the capability of RNNs to infer human preferences with simulated data.

Two recent studies have employed a similar workflow to identify time-varying variables using neural networks and simulation data. The first study (Schumacher et al., 2023) adopted a superstatistics framework to recover latent variable dynamics, which are governed by what they termed a "high-level transition model" and operate independently of the "low-level observation/cognitive model" responsible for processing observable data. Conversely, our current work focuses on trial-varying latent variables contingent upon cognitive models processing observable data. This focus is motivated by its applicability across various computational modeling frameworks. Despite this difference, our preliminary results in Appendix A1 suggest that LaseNet is also a good approach to capture trial-by-trial dependencies in models of the first kind (e.g., the non-stationary drift diffusion model proposed by Schumacher et al. (2023)). Nonetheless, further work is required to rigorously validate the robustness of this application.

The second study (Ghosh et al., 2024) focuses solely on state space models (i.e., HMMs), but does not demonstrate the applications to model families prevalent in cognitive modeling, such as RL models. Future work should compare LaseNet with the method proposed by Ghosh et al. (2024) directly on matched computational models and real datasets, as well as evaluate against various metrics for latent variable identification (Lueckmann et al., 2021).

### Limitations

Though LaseNet is generalizable and flexible for various computational models, there are some limitations. One limitation lies in its sensitivity to model misspecification; LaseNet presumes that the researcher has identified the "best" model for their empirical data. Consequently, its performance can substantially diminish when the true generative process differs from the cognitive model used during network training. Our results in Appendix C highlight this, showing a significant reduction in accuracy when fitting a four-state GLM-HMM to data from a three-state GLM-HMM. This underscores the necessity of performing robust model identification (Elsemüller et al., 2024; Rmus et al., 2024) prior to training LaseNet. We note that this sensitivity to misspecification is a challenge common to all model-fitting methods. Nevertheless, an intriguing avenue involves whether LaseNet could be trained to marginalize over model uncertainty, in the same way it marginalizes over model parameter uncertainty. To address this question, LaseNet could potentially be extended using a meta-learning framework (Hochreiter et al., 2001). By training on data generated from a distribution of models, LaseNet learns to adapt its inference process to the specific underlying cognitive model characteristics of the input data (the inner loop), guided by the overall meta-training across cognitive models (the outer loop). This would allow a single trained instance of LaseNet to more robustly infer time-varying latent variables. Crucially, to complement these efforts and ensure the validity of the outputs even when the true underlying model is not perfectly captured, quantifying the uncertainty associated with the inferred latent sequences is vital. As shown in Appendix D, evidential learning (Amini et al., 2020; Sensoy et al., 2018) provides one promising method for this uncertainty estimation. This will also be an important direction for future research.

In addition, LaseNet does not infer underlying model parameters that are relevant to the targeted latent variables. While it is less affected by models with many free parameters compared to standard estimators that rely on parameter recovery, understanding the relationship between model parameters and the resulting latent variables may still be critical for researchers. Therefore, parameter recovery with other neural-based estimators (Radev et al., 2020; Rmus et al., 2024) should be used in conjunction with LaseNet to provide a more comprehensive analysis.

Finally, while we tested our method in a real data set, we could not compare the inferred model variable directly with the ground truth, as this is, by definition, difficult to observe. We were able to demonstrate comparable qualitative results across methods and thus indirectly validate our approach. Future work should consider using a dataset that includes different measurement proxies for the latent states (e.g., reaction time (Botvinick et al., 2001), pupil size (Laeng et al., 2012)) and examine whether our proposed approach can outperform traditional statistical inference in real experimental settings.

## Conclusion

In this study, we present a new technique to extract latent cognitive model variables from observable behavior. LaseNet excels in handling computational models with intractable likelihoods, where traditional approaches are not applicable. The method is versatile, capable of identifying both discrete and continuous latent variables, and is generalizable across various computational models. Moreover, LaseNet is practical: it works with standard data sizes and can be applied at the individual participant level without high computational resources and technical expertise. Breaking down the barrier of intractable likelihood and recovering the latent dynamics of computational cognitive models will provide researchers with new insights into previously inaccessible dimensions in behavioral data.

An overview of the contents covered by the ensuing appendices:

A. “Appendix A Computational cognitive models”: including 4-P RL, Meta RL, HRL, Weber-imprecision, and GLM-HMM cognitive models used in this work.
B. “Appendix B Further experimental methods details”: including network training, computational cost, likelihood-dependent methods, and real-data experimental details.
C. “Appendix C Additional robustness tests”: experiments on model misspecification and trial length variation.
D. “Appendix D Uncertainty quantification”: example integration with evidential deep learning.

## Figures and Tables

**Fig. 7:**
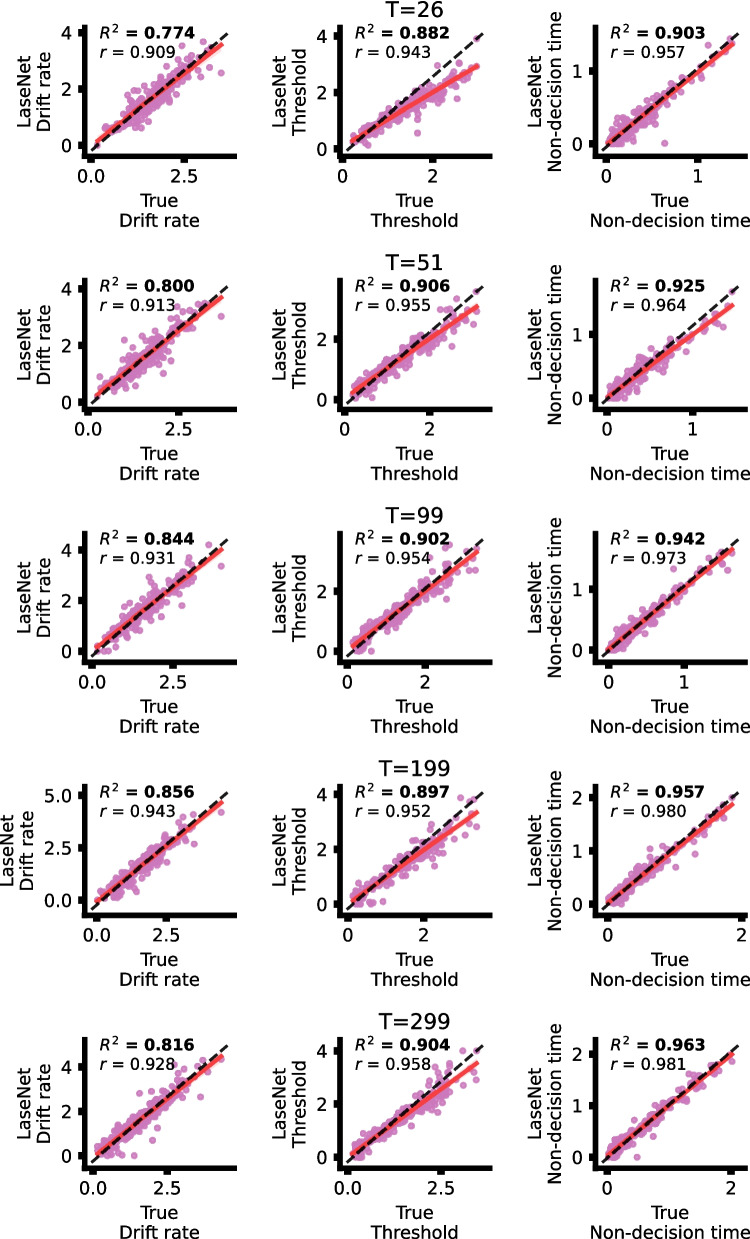
Non-stationary drift diffusion model (DDM): LaseNet infers trial-varying latent variables (drift rate, threshold, and non-decision time) from a non-stationary DDM (Schumacher et al., 2023). Each *row* represents a distinct trial T for illustration

**Fig. 8:**
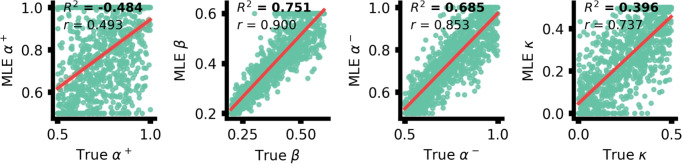
4-P RL parameters recovered by MLE $R^2$ is a R-squared score. *r* corresponds to the Pearson correlation coefficient, and the *red line* represents the least squares regression line. A high correlation score shows that MLE recovers well in this simple RL model

**Fig. 9:**
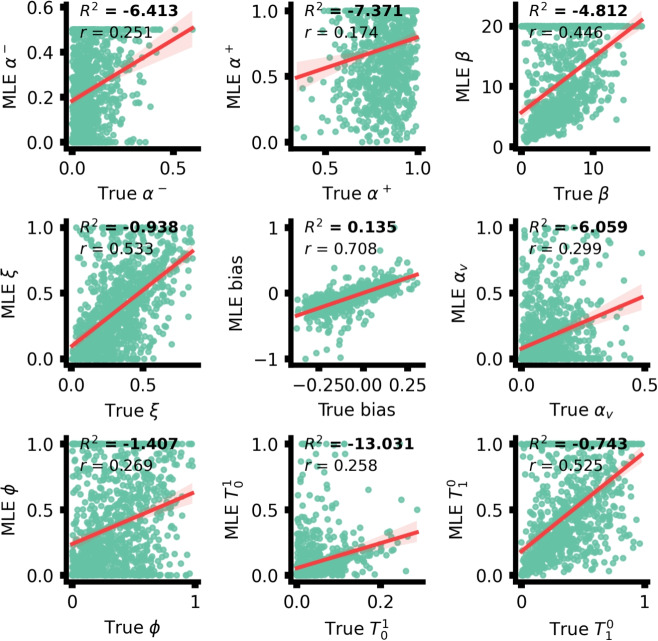
Meta RL parameters recovered by MLE. Unlike Fig. 8, MLE shows fairly limited parameter recovery in a highly parameterized model, which may impair MLE-dependent latent variable estimation

**Fig. 10:**
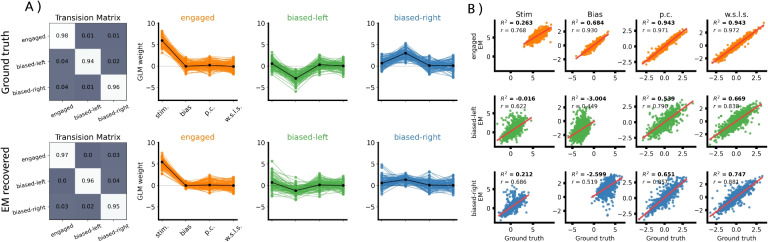
Three-state GLM-HMM recovered by EM. **A** *Top row*: simulated ground truth of transition matrix and GLM-weights in three states: engaged, biased-left, and biased-right. *Bottom row*: the well-recovered parameters from EM: high engaged state in the transition matrix, high stimulus GLM weight in the engaged state, negative bias weight in the left-biased state, and vice versa (GLM takes left as negative; right as positive value). **B** Each row represents GLM weights in three different states and each column represents four weighted features: stimuli, bias, previous choice, win-stay/loss-switch. In the GLM-HMM case, we showed that good recovery in parameters does not guarantee high precision in latent variables estimation compared to LaseNet (Fig. 4)

**Fig. 11:**
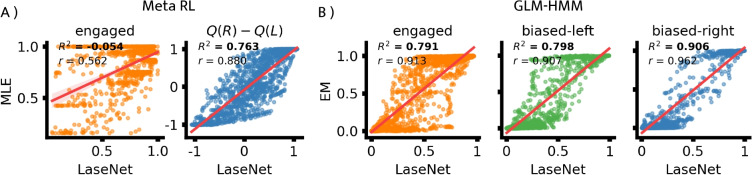
Correlation between LaseNet and likelihood-dependent methods in real data. **A** In Meta RL, MLE and LaseNet have similar estimated engaged probability and strong correlation in estimated Q-values difference. **B** In GLM-HMM, EM and LaseNet show high positive correlation in predicting state probability

**Fig. 12:**
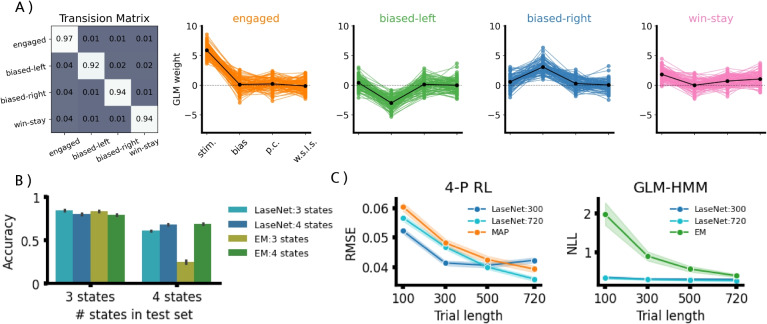
Robustness tests. **A** Four-state GLM-HMM with additional win-stay state compared to three-state GLM-HMM in Fig. 10. A win-stay has a relatively high win-stay, loss-switch weight in GLM. **B** Accuracy drops when fitting with a wrong model prior in both EM and LaseNet. A model identification is thus needed before inferring the latent states. **C** In both 4-P RL and GLM-HMM, LaseNet trained with longer trials exhibits more robust results across different trial lengths

**Fig. 13:**
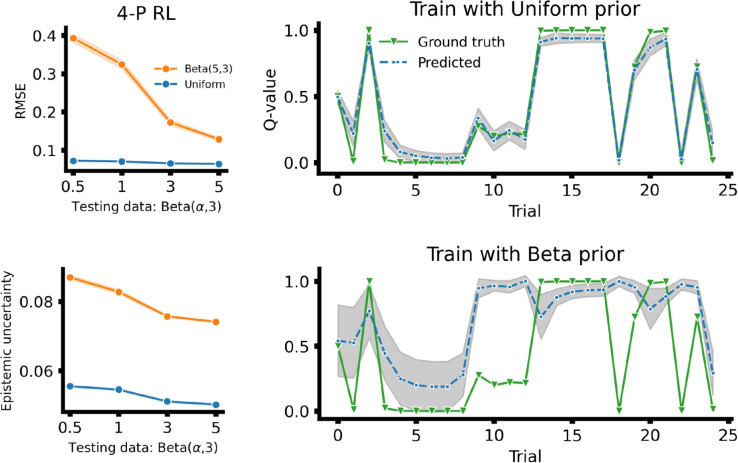
Uncertainty quantification in 4-P RL. Two LaseNet estimators: one is trained with a uniform prior and the other is trained with a beta prior (Fig 5A). LaseNet trained with a uniform prior has lower RMSE and uncertainty as testing with unseen data generated from different distributions. In right columns, grey area shows $\pm 1$ standard deviation

## Data Availability

The raw data studied in this paper are publicly available. The dynamic foraging dataset can be accessed at  https://datadryad.org/stash/dataset/doi:10.5061/dryad.cz8w9gj4s. The IBL mice dataset can be accessed at  https://doi.org/10.6084/m9.figshare.11636748.

## Appendix A Computational cognitive models

### Four-parameter reinforcement learning model

On each trial t, the four-parameter reinforcement learning model (4P-RL) includes a stickiness parameter $\kappa$ which captures the tendency to repeat choice from the previous trial:

9 $$\begin{aligned} P(a) \propto exp(\beta Q + \kappa \ \text {same}(a,a_{t-1})) \end{aligned}$$

Once the reward *r* has been observed, the action values are updated based on 4. In addition, we adopt a counterfactual updating in this model, where the value of the non-chosen action also gets updated on each trial (Eckstein et al., 2022):

10 $$\begin{aligned} \begin{gathered} \delta _\text {unchosen} = (1-r) - Q_{t}(1-a) \\ Q_{t+1}(1-a) = Q_{t}(1-a) + \alpha \,\delta _\text {unchosen} \end{gathered} \end{aligned}$$

Instead of having one learning rate, the model differentiates between positive and negative feedback (Niv et al., 2012), by using different learning rates - $\alpha ^+$ and $\alpha ^-$ for updating action values after positive and negative outcomes, respectively:

$$\begin{aligned} Q_{t+1}(a)&= {\left\{ \begin{array}{ll} Q_{t}(a) + \alpha ^+\,\delta \  & \text{ if } r\ > 0 \\ Q_{t}(a) + \alpha ^-\,\delta \  & \text{ if } r\ \le 0 \end{array}\right. } \end{aligned}$$

The 4P-RL model thus includes four free parameters: positive learning rate ($\alpha ^+$), negative learning rate ($\alpha ^-$), softmax beta ($\beta$) and stickiness ($\kappa$). As simulating data, all parameter priors are drawn from $\mathcal {U}(0,1)$, except for $\beta$, which is drawn from $\mathcal {U}(0,10)$.

### Meta reinforcement learning model with dynamic noise

The meta-learning model was first proposed by Grossman et al. (2022). Li et al. (2024) extended the model with dynamic noise and shows that the modified model fits the experimental data better. The model assumes that a participant has two latent states with time-varying transition probabilities ($T_0^1$, $T_1^0$), which denote transitions from the random state to the engaged state and vice versa. In the random state, the decision policy is random and uniform. In the engaged state, on each trial t, a decision is sampled from choice probabilities obtained through a softmax function applied to the action values of the left and right actions with a bias. The probability of the left choice is given by:

11 $$\begin{aligned} P_t(l)=\frac{1}{1+exp(\beta \times \left( Q_t(r)-Q_t(l) \right) +bias)} \end{aligned}$$

Regardless of the state that a participant is in, once the reward is observed, assuming the left action is chosen, Q-values are updated as follows:

12 $$\begin{aligned} Q_{t+1}(l)=Q_t(l)+\alpha _{t}\cdot \delta _t\cdot \left( 1-E_t \right) \end{aligned}$$

where $\alpha _{t}$ is either $\alpha _{+}$ or $\alpha _{-}$ based on positive or negative outcomes, respectively, $E_t$ is an evolving estimate of expected uncertainty calculated from the history of absolute reward prediction errors (RPEs):

13 $$\begin{aligned} \begin{gathered} E_{t+1} = E_t + \alpha _v\cdot v_t \\ v_t=\left| \delta _t \right| -E_t \end{gathered} \end{aligned}$$

When RPE is negative, the negative learning rate is dynamically adjusted and lower-bounded by 0:

14 $$\begin{aligned} \alpha _{(-)_{t}} = max(0, \,\, \psi \cdot (v_t+\alpha _{(-)_{0}}) + (1-\psi )\cdot \alpha _{(-)_{t-1}}) \end{aligned}$$

Lastly, the unchosen action is forgotten:

15 $$\begin{aligned} Q_{t+1}(unchosen\,\,action) = \xi \cdot Q_{t}(unchosen\,\,action) \end{aligned}$$

The model thus has nine parameters: two transition probabilities ($T_0^1$, $T_1^0$), softmax beta ($\beta$), bias(for the right action), positive learning rate ($\alpha ^+$), baseline negative learning rate ($\alpha ^-_0$), learning rate of RPE magnitude integration ($\alpha _v$), meta-learning rate for unexpected uncertainty ($\psi$), and forgetting rate ($\xi$). As simulating data, all parameter priors are drawn from $\mathcal {U}(0,1)$, except that $\beta$ is from $\mathcal {U}(0,20)$

Note that the likelihood is tractable in Meta RL, because the model assumes that the latent state only affects the policy to choose an action. In the random state, information is thus still being processed (e.g., Q-values updating given rewards) (Li et al., 2024).

### Hierarchical reinforcement learning

The hierarchical reinforcement learning (HRL) model follows a similar updating policy as 4-P RL except that the model tracks the values of each arrow-following rule. With N arrows, the complete probability of choosing each arrow is given by:

16 $$\begin{aligned} P(arrow_i) = \frac{\exp (\beta \;Q_{t}(arrow_i)}{\sum _{i = 1}^{N} \exp (\beta \;Q_{t}(arrow_i))} \end{aligned}$$

Once the agent selects an arrow, it greedily chooses the direction based on the side (left or right) indicated by the arrow. For simplicity, different from 4-P RL, the model has no stickiness $\kappa$ and has a single shared learning rate for both positive or negative outcomes. The model includes only two free parameters: learning rate $\alpha$ $\sim \mathcal {U}(0.4, 0.7)$; and softmax beta $\beta$ $\sim \mathcal {U}(1, 10)$.

#### HRL likelihood is intractable

The log-likelihood in the HRL model can be formally written as such:

17 $$\begin{aligned} \mathcal {L}(\theta ) = \sum _{t=1}^{T} \log P(a_t \mid \textbf{x}_{1:t}, \theta ) \end{aligned}$$

where *T* is the total number of trials, $a_t$ denotes the action chosen by the participant at trial *t* (e.g., left/right), and $\textbf{x}_{1:t} = (x_1, \ldots , x_t)$ represents the history of observable task variables, including stimuli presented, actions previously taken, and rewards received up to trial *t*.

To calculate $P(a_t \mid \textbf{x}_{1:t}, \theta )$, we marginalize over all possible sequences of latent states $\textbf{z}_{1:t} = (z_1, \ldots , z_t)$ up to trial *t*. Let $\mathcal {Z}$ be the set of *M* distinct values any single latent state $z_t$ can be (e.g., *M* rules or ’arrows’). The set of all $M^t$ possible latent state sequences of length *t* is denoted by $\mathbb {Z}_t = \{ (z'_1, \ldots , z'_t) \mid z'_k \in \mathcal {Z} \text { for } k=1, \ldots , t \}$. The log-likelihood can then be expressed as:

18 $$\begin{aligned} \mathcal {L}(\theta ) = \sum _{t=1}^{T} \log \left( \sum _{\textbf{z}_{1:t} \in \mathbb {Z}_t} P(a_t, \textbf{z}_{1:t} \mid \textbf{x}_{1:t}, \theta ) \right) \end{aligned}$$

where the inner summation is over all possible latent state sequences $\textbf{z}_{1:t}$ of length *t*. We can further expand the probability of each sequence as below:

19 $$\begin{aligned} P(a_t, \textbf{z}_{1:t} \mid \textbf{x}_{1:t}, \theta ) = p(z_1)\,p(a_1 \mid z_1, x_1, \theta ) \prod _{s=2}^{t} p(z_s \mid z_{s-1})\,p(a_s \mid z_s, x_s, \theta ) \end{aligned}$$

where $z_s$ is the chosen unobservable arrow/rule at trial s. This likelihood is computationally intractable because, for each trial *t*, calculating $P(a_t \mid \textbf{x}_{1:t}, \theta )$ requires summing overall $M^t$ possible latent state sequences $\textbf{z}_{1:t}$. For instance, if there are $M=3$ possible latent states (rules/arrows), computing the sum for $P(a_2 \mid \textbf{x}_{1:2}, \theta )$ involves $3^2=9$ sequences (e.g., $(z_1=1, z_2=1), (z_1=1, z_2=2), \ldots , (z_1=3, z_2=3)$). The number of terms in this summation grows exponentially with *t*. Consequently, for a realistic number of trials *T*, direct computation by enumerating all $M^T$ paths for the final terms (or $M^t$ for intermediate terms) is infeasible.

### Weber-Imprecision

The Weber-imprecision model, a variant of the Bayesian generative model (Findling et al., 2021), assumes that participants form posterior beliefs B(t) about the correct mapping at each trial t. Here, the term “latent state” refers to the mapping tracked by participants. To model action selection, we applied the softmax rule to the logarithm of these beliefs:

20 $$\begin{aligned} P_i(t) \sim \exp (\beta \cdot \ln B_i(t)) \end{aligned}$$

where $P_i(t)$ denotes the probability of choosing action *i* in trial *t*, $B_i(t)$ is the belief that action *i* is the correct action in trial *t* (calculated by marginalizing over latent state beliefs) and $\beta$ is the inverse temperature parameter. Unlike the sparse reward environment in Findling et al. (2021), our setup delivered rewards $r_t$ for correct actions in every trial. The task involved three stimuli and four actions, resulting in 24 possible mappings.

In volatile environments, the Weber-Imprecision model adapts to changes in the correct mapping by adding noise with a probability $\epsilon _t$ described in “Intractable models”. When the current latent state changes between trial $t-1$ and *t*, the new latent state $z_t$ is drawn from the set of potential latent states $\{1, \dots , K\}$ according to a multinomial distribution with probabilities $\varvec{\gamma } = \{\gamma _1, \dots , \gamma _K\}$, where K is 24.

21 $$\begin{aligned} z_t \mid z_t \ne z_{t-1} \sim \text {Multinomial}(\varvec{\gamma }) \end{aligned}$$

with $\sum _{k=1}^{K} \gamma ^k = 1$ and excluding the preceding state. The priors probability over $\gamma$ is uninformative and follows a flat Dirichlet distribution. The model includes three free parameters: $\mu$ $\sim \mathcal {N}(0.05, 0.003)$, $\lambda$ $\sim \mathcal {N}(1.22, 0.06)$, and $\beta$ $\sim \mathcal {N}(5, 0.023)$.

Due to the sequential nature of the belief updating, and the need to marginalize over latent states, which are probabilistically updated in an unobservable way, the likelihood function for the Weber imprecision model is computationally intractable.

### Hidden Markov models with Bernoulli generalized linear model observation

We use a framework based on HMMs with Bernoulli generalized linear model (GLM) observations to analyze decision-making behaviors in mice (Ashwood et al., 2022). The resulting GLM-HMM, also known as an input-output HMM, supports an arbitrary number of states that can persist over a large number of trials and have different dependencies on the stimulus and other covariates. A GLM-HMM consists of two basic components: an HMM that governs the distribution across latent states and a set of state-specific GLMs, specifying the strategy employed in each state. For a GLM-HMM with K latent states, the HMM has a $K\times K$ transition matrix *A* specifying the probability of state transition,

22 $$\begin{aligned} P(z_t = k \mid z_{t-1} = j) = A_{jk} \end{aligned}$$

where $A_{jk}$ denotes the transition from state *j* to state *k*, $z_{t-1}$ and $z_t$ indicate the latent state at trials $t-1$ and *t*, respectively. To represent the state-dependent mapping from inputs to decisions, the GLM-HMM comprises K independent GLM weights, each defined by a vector *w* indicating how inputs are integrated in that particular state. We use four-dimensional *w* for inputs: stimuli, bias, previous choice, and win-stay/lose-switch. With a three-state GLM-HMM, there are 21 free parameters: $\mathbf {\theta } \equiv \left\{ A, {w}_{k=1}^{k=3} \right\}$, which *A* is a $3\times 3$ transition matrix and $w_k \in \mathbb {R}^4$ is a GLM weight vector for state *k*.

Similar to HRL, the GLM-HMM faces the challenge of computing likelihoods by marginalizing over all possible sequences of *K* latent states across *T* trials. This marginalization over the $K^T$ potential paths quickly becomes computationally intractable as the number of trials increases.

### Non-stationary drift diffusion model

The latent variables investigated previously are conditioned by observable variables (e.g., rewards, actions) and the employed cognitive model. In contrast, a distinct class of models involves latent variables that evolve independently of these observational inputs and the cognitive model. In such cases, the generative process for a latent variable takes the form $z_t\sim f(z_{t-1}, \theta _f)$, where *f* is a transition model parameterized by $\theta _f$, governing the evolution from the prior state $z_{t-1}$.

The non-stationary drift-diffusion model (DDM) proposed in Schumacher et al. (2023) exemplifies this latter type. The non-stationary DDM assumes that the history of past choices influences current cognitive processes. Inspired by the superstatistics framework (Metzner et al., 2021), it incorporates a stateful Gaussian process to describe the trial-by-trial dynamics of DDM parameters (i.e., drift rate, threshold, and non-decision time). Here, we applied LaseNet to infer the trial-varying latent variables in the non-stationary DDM. We first simulated agents (400 trials per agent) with the open source code provided in Schumacher et al. (2023). LaseNet was then trained with 3000 simulated agents and tested on 200 distinct simulated agents. Preliminary results (Fig. 7) demonstrate the promising potential of LaseNet for this category of latent variable inference.

## Appendix B Further experimental methods details

### ANN training specification

**Table 2:** Hyperparameters selection

Hyperparameter	Sweep range
# of units in RNN layer	$\mathcal {U}(36, 326)$
dropout rate in RNN layer	$\mathcal {U}(0.05, 0.25)$
dropout rate in 1st MLP layer	$\mathcal {U}(0.01, 0.1)$
dropout rate in 2nd MLP layer	$\mathcal {U}(0.01, 0.05)$
learning rate	0.003 or 0.0003

**Table 3:** Performance comparison of bidirectional versus unidirectional GRU architectures on two intractable models. The bidirectional architecture demonstrated superior performance, indicated by higher accuracy (lower Log Loss for discrete variables and lower RMSE for continuous variables), in most contexts. Metrics are reported as mean ± standard deviation across 3000 testing samples. Lower mean values, signifying better performance, are highlighted in bold

**Models**		**HRL**		**Weber-imprecision**	
**Training Samples**	**Direction**	**Log Loss**	**RMSE**	**Log Loss**	**RMSE**
600	Bi-Direction	**0.17±0.148**	0.14±0.076	0.81±0.389	0.12±0.035
	Uni-Direction	0.25±0.125	0.14±0.079	**0.80±0.413**	**0.11±0.037**
1000	Bi-Direction	**0.16±0.141**	**0.13±0.075**	**0.65±0.343**	0.11±0.035
	Uni-Direction	0.24±0.122	0.14±0.079	0.70±0.365	0.11±0.041
3000	Bi-Direction	**0.16±0.146**	0.13±0.082	**0.58±0.298**	**0.11±0.028**
	Uni-Direction	0.23±0.115	0.13±0.080	0.59±0.320	0.12±0.037
6000	Bi-Direction	**0.16±0.145**	**0.12±0.083**	**0.52±0.269**	**0.11±0.030**
	Uni-Direction	0.23±0.114	0.13±0.082	0.55±0.284	0.12±0.035

**Table 4:** Training time ($\text {mean} \pm \text {std. dev.}$ in minutes) of LaseNet on two likelihood-intractable models that predict both continuous and discrete latent variables. Each LaseNet was trained with a learning rate of $3e-4$ and a batch size of 256 on the L4 GPU provided by Google Colab

**Models**	**HRL**				**Weber-imprecision**			
**# of Trials**	**720**				**300**			
**# of Samples**	**600**	**1000**	**3000**	**6000**	**600**	**1000**	**3000**	**6000**
Mean	4.25	6.28	17.36	28.44	2.72	3.51	7.84	12.02
Std. Dev	0.015	0.011	0.017	2.2	0.21	0.3	0.82	0.97

### Details of likelihood-dependent methods

In this appendix, we described in detail the likelihood-dependent method to infer latent variables. The fitting process consists of two steps: identifying the best-fitting parameters and then inferring the latent variables with the best-fitting parameters. Here, we used the coefficient of determination $R^2$ and the Pearson correlation coefficient *r* to measure the performance of parameter recovery. $R^2$ represents the proportion of variance in true parameters that can be explained by a linear regression between true and predicted parameters. The best possible $R^2$ score is 1. Pearson correlation coefficients *r* represent the strength of a linear association between true and predicted parameters. Since a positive correlation is desired, the best possible *r* score is 1.

#### Maximum likelihood and maximum a posteriori estimation

Maximum likelihood estimation (MLE) leverages probability theory and estimation of likelihood $P(Y\mid \theta )$ of the data given the model parameters and assumptions (Myung, 2003). The best-fitting parameters are determined by maximizing the log-likelihood of the data:

23 $$\begin{aligned} \theta _{MLE} = \underset{\theta }{\text {arg}\,\,\text {max}} P(Y \mid \theta ) = \underset{\theta }{\text {arg}\,\,\text {max}} \sum _{i} \text {log}\, P(y_{i}\mid \theta ) \end{aligned}$$

To find the parameters via MLE, we used the sequential quadratic programming (SQP) method provided by MATLAB fmincon. SQP allows for solving the optimization problem with constraints. The constraints we imposed on are the empirical ranges of each model parameter (e.g., [0, 1] for parameters’ search space). Figures 8 and 9 show the recovered parameters in experiments with tractable models. With the recovered parameters and the observable data *Y*, we then derived the latent variables such as Q-values by running through the RL functions.

Maximum a posteriori estimation (MAP) relies on a similar principle, with the addition of a prior $P(\theta )$ to maximize the log posterior:

24 $$\begin{aligned} \theta _{MAP} = \mathop {\mathrm {arg\,max}}\limits _{\theta } \sum _{i} \,[\,\text {log}\, P(y_{i}\mid \theta ) + \text {log}\,P(\theta )\,] \end{aligned}$$

We employed MAP in prior misspecification experiments 3.4 for fitting data generated from a 4-P RL model. Specifically, we tested different $\beta$ parameters prior (Fig. 5, keeping other priors with uniform distributions. Same as MLE, we used the SQP algorithm in MATLAB and derived latent variables with the recovered parameters and the observable data.

#### Sequential Monte Carlo

Sequential Monte Carlo (SMC) methods (Gordon et al., 1993) approximate probability distributions with weighted particles that evolve as new observations arrive, enabling inference in nonlinear, non-Gaussian dynamic systems. The particle weights reflect likelihood, providing an estimate of the posterior distribution and its uncertainty (Samejima et al., 2003). In our study, the posterior distribution is $P(Z \mid Y_{1:t}, \theta )$, where *Z* denotes targeted latent variables (particles), $Y_{1:t}$ denotes the observable variables from trial 1 to t, and $\theta$ are known model parameters. In the HRL model, we applied particle filtering (PF) algorithms to recover the latent arrows/cues. Specifically, the PF consists of procedures:

1. Propagate: particles are propagated based on the probability of choosing each arrow (Appendix  A).
2. Update: particle weights are updated based on the agent’s actions and the associated Q values. For instance, if the agent selects the unique arrow, the particle weight corresponding to that arrow is set to 1, while all others are assigned a weight of 0. Conversely, if the agent chooses a non-unique arrow, the weights are adjusted proportionally to the Q values.
3. Resample: if the $\text {effective sample size (ESS)} = 1/\sum w_i^2$ (Liu & Chen, 1995) is less than half of the number of particles, particles are resampled based on the weights.

We implemented the algorithm by iterating step 1 to step 3 across 720 trials and using a total number of 1000 particles. Each particle is initialized randomly from a uniform distribution.

In benchmarking the Weber-imprecision model, we employed the same method used in Findling et al. (2021): $\text {SMC}^2$, an algorithm that extends from iterated importance sampling (Chopin, 2002) and particle Markov chain Monte Carlo method (Doucet et al., 2000). We used a total number of 51200 particles over 300 trials.

#### Expectation-Maximization

The expectation-maximization (EM) algorithm is an iterative method for determining the maximum likelihood or maximum a posteriori estimates of parameters in statistical models based on unobserved latent variables *Z*. The log-likelihood to be maximized is thus given by marginalizing out latent variables *Z*:

25 $$\begin{aligned} \text {log}\,P(Y\mid \theta ) = \sum _{z} \text {log}\,P(Y, Z \mid \theta ) \end{aligned}$$

Nonetheless, this is intractable in the GLM-HMM model as described in 7.5. Hence, following the same approach in Ashwood et al. (2022), we maximize the complete log likelihood instead. During the E-step of the EM algorithm, we compute the expected complete data log-likelihood (ECLL), which is a lower bound on Eq. 25. We can derive the lower bound as:

$$\begin{aligned} \text {log}\,P(Y\mid \theta )&= \text {log}\,\sum _{z} P(Y, Z \mid \theta ) \\&= \text {log}\,\sum _{z} P(Z \mid Y, \theta )\,\,\frac{ P(Y, Z \mid \theta )}{P(Z \mid Y, \theta )} \\&= \text {log}\left( \mathbb {E}_{P(Z\mid Y, \theta )}\left[ \frac{ P(Y, Z \mid \theta )}{P(Z \mid Y, \theta )} \right] \right) \\&\ge \mathbb {E}_{P(Z\mid Y, \theta )}\,\,\text {log}\left[ \frac{ P(Y, Z \mid \theta )}{P(Z\mid Y, \theta )} \right] \end{aligned}$$

Then, during the ‘maximization’ or M-step of the algorithm, we maximize the ECLL with respect to the model parameters $\theta$. To understand how this iterative algorithm converges to the desired log-likelihood, please see Wu (1983) for the actual proof.

Figure 10 shows the recovered transition matrix and GLM weights in a three-state GLM-HMM model after applying EM. We then infer the most probable latent states by running the E-step with the best-fitting parameters and the input data.

### Infer latent variables in mice dataset

In this section, we outlined the training details of LaseNet for real mice data.

#### Dynamic foraging dataset

We first simulated 9000 participants from Meta RL model described in Appendix A; each simulated participant has 720 trials. The parameters of Meta RL model were drawn from the empirical distribution given by  (Li et al., 2024). We then trained LaseNet with the simulated participants; LaseNet takes a time series of rewards and actions as input (Table 1), and outputs three time-varying latent variables: attentive states, Q-values of leftward action, and Q-values of rightward action. Compared to MLE, we found higher correlation in Q-values difference estimation than latent state probability estimation (Fig. 11A).

#### IBL dataset

We simulated 6000 participants from the GLM-HMM model described in  7.5; each simulated participant has 500 trials. We used an equal transition probability between states. GLM weights were drawn from the empirical distribution given by (Ashwood et al., 2022). We trained LaseNet with simulated participants. LaseNet takes a time series of rewards, actions, and stimuli as input (Table 1), and outputs one latent variable with three possible states:engaged, left-biased and right-biased. Figure 11B shows a high correlation between EM and LaseNet in inferring latent state probabilities.

## Appendix C Additional robustness tests

### Model misspecification

We tested model misspecification by fitting data generated from a three-states GLM-HMM to estimators with a four-states prior, and vice versa. For LaseNet estimators, we trained two neural networks with data generated from three and four states priors, respectively. Data generated from a three-states GLM-HMM has the same model prior as previous experiments (Fig. 10A), while data from a four-states GLM-HMM has the prior shown in Fig. 12A. We added a "win-stay" state for a four-states GLM-HMM based on empirical results in (Ashwood et al., 2022). Each LaseNet estimator was trained with 3000 simulated samples. We generated an additional 500 simulated samples for each state priors as test sets. All samples have 720 trials. We compared our trained LaseNet estimators with EM having either three or four states priors. We found that both EM and LaseNet have lower accuracy when fitting with misspecified models (Fig. 12B). Hence, model identification should be performed before inferring latent states.

### Trial length variation

We examined LaseNet in inferring latent states with different trial lengths, because in real experiments, researchers commonly collect varying trial lengths. We tested two cognitive models, 4-P RL and GLH-HMM, with four different trial lengths lengths: 100, 300, 500, and 720. In comparison to likelihood-dependent methods, we trained two LaseNet estimators with data generated from 300 and 720 trials, respectively. Each LaseNet estimator was trained with 22,000 simulated samples for 4-P RL and with 9000 simulated samples for GLM-HMM. For testing, we generated an additional 500 simulated samples for each trial length. Figure 12B shows that training with 720 trials reaches higher precision consistently across all trial lengths compared to likelihood-dependent methods. Note that EM is susceptible to a short trial length because we can only approximate the likelihood for GLM-HMM as described in 8.2.3; shorter trial length yields fewer data points for approximation.

## Appendix D Uncertainty quantification

In this appendix, we explored the potential of integrating with evidential deep learning (Amini et al., 2020; Sensoy et al., 2018). Evidential deep learning aims to train a neural network to learn a higher-order, evidential distribution and then output the hyperparameters of the evidential distribution. Here, we adopted the loss functions proposed in Amini et al. (2020) to quantify the uncertainty for inferring Q-values in a 4-P RL model. The network structure is the same as Fig. 2 except that the objective function is maximizing and regularizing evidence. To measure the effect of evidential deep learning, we compared two LaseNet estimators trained with different $\beta$ model priors:uniform and beta distribution, illustrated in Fig. 5A. Each LaseNet estimator was trained using 3000 simulated samples, each consisting of 500 trials. Figure 13 shows that the LaseNet estimator trained with a uniform prior exhibits lower RMSE and uncertainty. Higher uncertainty also corresponds to higher RMSE. Our implementation is extended from: https://github.com/aamini/evidential-deep-learning

## Code availability

We used Tensorflow for all neural networks. Code to reproduce results is available at  https://github.com/ti55987/lasenet.
